# Therapeutic Approaches Using Host Defence Peptides to Tackle Herpes Virus Infections

**DOI:** 10.3390/v1030939

**Published:** 2009-11-18

**Authors:** Håvard Jenssen

**Affiliations:** Department of Science, Systems & Models, Roskilde University, Universitetsvej 1, Building 18.1, DK-4000 Roskilde, Denmark; E-Mail: jenssen@ruc.dk; Tel.: +45 4674 2877; Fax: +45 4674 3010

**Keywords:** cationic host defence peptides, antiviral therapy, herpes, antimicrobial peptides, innate defense regulators, immune stimulation

## Abstract

One of the most common viral infections in humans is caused by herpes simplex virus (HSV). It can easily be treated with nucleoside analogues (e.g., acyclovir), but resistant strains are on the rise. Naturally occurring antimicrobial peptides have been demonstrated to possess antiviral activity against HSV. New evidence has also indicated that these host defence peptides are able to selectively stimulate the innate immune system to fight of infections. This review will focus on the anti-HSV activity of such peptides (both natural and synthetic), describe their mode of action and their clinical potential.

## 1. Introduction

Introduction of the therapeutic use of penicillin during World War II followed by the discovery and development of other antibiotics targeting bacterial, and later also viral and fungal pathogens, have had a tremendous impact on human life expectancy. However, infectious diseases remain the main cause of morbidity and mortality accounting for nearly one-third of the global deaths annually [1]. In addition to this, there have been reports of alarming increases in the prevalence of drug-resistant clinical viral strains, especially in cases related to HIV, highlighting the urgent need for new antiviral intervention strategies. Despite these facts, there has been a decline in the number of new drugs that has been introduced to the market, and the pharmaceutical industry has largely withdrawn from the field of anti-infective discovery and development. In parallel, the academic interest in the field has bloomed, leading to new drug strategies for microbial intervention, e.g., antibodies and small molecule agonists and antagonists, and the success of these ventures has been reviewed elsewhere [2–7]. Another very promising strategy is tailored around the so-called host defence peptides (HDPs; also called cationic antimicrobial peptides), and this is currently being pursued by several small and mid-sized biotechnology companies. In large, this review will discuss HDPs and their inhibitory activity against herpes simplex virus (HSV), in addition to some promising peptides in clinical trials, supporting the idea that derivatives of HDPs may one day serve as a valid choice for HSV intervention.

## 2. Tailoring antiviral peptide drugs

Numerous strategies can be envisioned for the design of peptide drugs that are able to inhibit herpes simplex virus infection. For example, one can create peptides that directly target the virus particle (*i.e.,* viral envelope or glycoproteins), or one can design antiviral peptides that target the host cell (*i.e.,* blocking viral attachment and/or more specific entry receptors) through peptide-protein interaction. A third strategy would be to create peptides that could stimulate the host innate immune system to oppose and control the viral infection.

**Figure 1.**
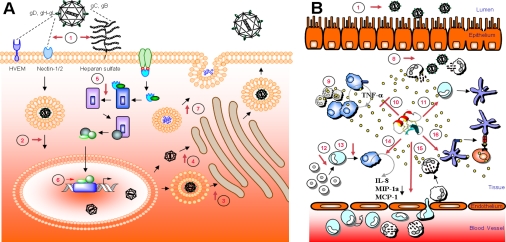
Known properties of antiviral/immunomodulatory host defense peptides. The figure illustrates possible targets in a single cell **(A)** and at the systemic level **(B)**. HDPs may contribute their antiviral activity through interference with viral attachment and entry **(1)**. The viral envelope contains more than a dozen viral glycoproteins and five of them (gB, gC, gD, gH and gL) have been shown to participate in viral entry. Binding of virus to the cell is mediated by the binding of gB and/or gC to heparan sulfate chains on the cell surface proteoglycans. This facilitates the binding of gD to one of its cell surface receptors: HVEM, nectin-1, nectin-2, or specific sites on heparan sulfate generated by 3-O-sulfotransferases. Binding of gD to any one of these receptors triggers fusion of the viral envelope with the cell membrane. This membrane fusion requires the action of gB and a gH-gL heterodimer as well as gD and the gD receptor. Transport of HSV though the cytoplasm to the nucleus may also be targeted **(2)**. The HSV capsid buds through the inner nuclear membrane forming an enveloped virion particle. Egress of virions from host cell may occur by either of the two general pathways; either budding through the outer nuclear membrane and vesicular transport through the Golgi apparatus to the exterior of the cell **(3)** or de-enveloping of the capsid through the nuclear membrane and capsid budding into the Golgi apparatus, forming an enveloped virion, which is transported to the surface by vesicular movement **(4)**. The virus may also be targeted indirectly through HDP stimulation or suppression of cellular signaling cascades **(5)**, interference with gene transcription **(6)** or alteration of different effector molecules produced by the cells and/or degranulation **(7)**. Antiviral responses to HDP treatment may also have systemic effects, as it is well established that high concentrations of HDPs are released from activated neutrophil secretory vesicles **(8)**. HDPs like LL-37 can also alter TLR-induced responses reducing pro-inflammatory mediators **(9)** and inhibit apoptosis of neutrophils **(10)**. HDP may also trigger differentiation of dendritic cells **(11)** and monocytes to macrophages **(12, 13)**. HDP-activated macrophages are also known to release several chemokines **(14)** promoting recruitment of leukocytes into the afflicted area **(15)**. HDPs like LL-37 also promote expression of co-stimulatory molecules on dendritic cells leading to expression of T_h_1 cytokine IL-12 **(16)**.

Human herpes simplex virus comes in two flavors, type 1 or type 2 (HSV-1 and HSV-2), and they are the primary agents of recurrent facial and genital herpetic lesions, respectively [8,9]. They are also the two most widely studied human herpes viruses, leaving a very detailed picture of how these viruses infect and replicate in the host cells. In brief, initiation of HSV infection involves attachment of viral glycoprotein C (gC) and/or glycoprotein B (gB) to heparan sulfate on the host cell surface (Figure 1) [10,11]. Although interaction with heparan sulfate can be viewed as somewhat unspecific, it is well recognized that heparan sulfate functions as an attachment receptor for both HSV-1 and HSV-2 [12], explaining the low HSV infectivity of cells deficient in heparan sulfate expression [10,13,14]. However, viral attachment to heparan sulfate does not automatically enable viral entry. The entry process requires viral glycoprotein D (gD) interaction with one or several co-receptor molecules on the host cell surface [15], *i.e.,* (1) HveA/HVEM (herpes virus entry mediator) [16,17], (2) nectin-2α/HveB/PRR2/, nectin-1α/HveC/PRR1 (poliovirus receptor related immunoglobulin) [17,18] and nectin-1β/HIgR (herpes immunoglobulin-like receptor) [19] and (3) 3-O-sulfated heparan sulfate (3-O-HS) [20,21] (Figure 1). The viral envelope then fuses with the host cell membrane with the help of viral gB, gD, and a heterodimer of gH and gL, resulting in release of the viral tegument proteins and the viral capsid into the cytosol. After the initial infection and a successful replication cycle, the viral progeny can be released either through host cell lysis, exocytosis or it can be transferred across cell junctions to the neighboring cell (cell-to-cell spread). The latter process is not fully understood, though it is clear that by performing cell-to-cell spread, the virus avoids neutralizing antibodies and other elements of the hosts’ immune system [22]. Despite the limited knowledge currently available about this process, viral mutants deficient in expressing gE or gI, are also significantly suppressed in their ability to produce plaques [22,23], supporting the hypothesis that gE and gI mediate HSV transfer across cell junction by interactions with cell junction components [24].

Besides targeting the viral particle or the viral attachment and entry mechanism, another valid but ambitious strategy would be to target the host innate immune system for preventing and curing an infection. This strategy has been made possible through the discovery of specific pathogen recognition receptor families such as the Toll-like receptors, peptidoglycan recognition proteins and intracellular sensors of microbial components, e.g., the Nod-like receptors and the retinoic acid-inducible gene-I-like receptors [25,26]. The innate immune system has evolved through evolution and is intrinsically conserved and highly efficient, considering the relative infrequency in which infectious diseases occur, thus rendering it a very suitable target for the development of novel antiviral drugs. An excellent example of an approved immunemodulator for treatment of viral infections are the cytokine-based recombinant and modified forms of interferon-α (e.g., pegylated IFN-α), currently primarily being used for chronic hepatitis infections, but also being administered in severe cases of herpes-associated diseases in immunosuppressed patients. Another immune stimulant, Isoprinosine, enhances T cell proliferation and activity, and is also approved for treatment of HSV, though it is significantly less active than other more traditional herpes drugs [27]. Despite this increased understanding of the antiviral immune mechanisms and the success of pegylated IFN-α and Isoprinosine, a very limited number of drugs have been approved for medical use against HSV in the last few decades. Hence, the current trends for HSV treatment is still mainly targeting the viral replication process with Acyclovir [28] or synthetic analogues thereof [29].

## 3. Host defence peptides

**Figure 2.**
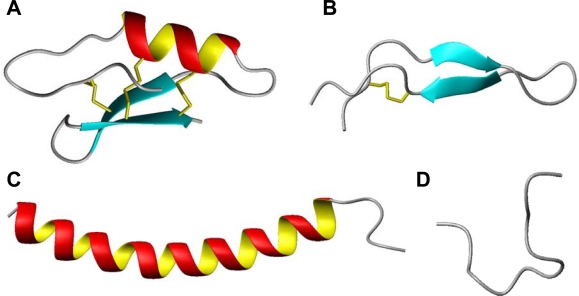
Structural classes of antimicrobial peptides; **(A)** mixed structure of Plectasin, a defensin-like molecule (PDB code 1ZFU) [44], **(B)** β-looped lactoferricin (PDB code 1LFC) [45], **(C)** α-helical human cathelicidin LL-37 (PDB code 2K6O) [46], **(D)** extended indolicidin (PDB code 1G89) [47]. The figures have been prepared with use of the graphic program MolMol 2K.1 [48].

Evolution has resulted in a finely tuned defence system relying on the interplay between the germline-encoded innate immunity and antigen-specific adaptive immunity mechanisms, a process which is known to involving a specter of different cytokines. Recently it has been recognized that many host defence peptides are able to modulate these cytokine levels, reinforcing their role as regulators of innate immune and inflammatory responses of mammals, amphibians and insects [30–32]. Being such fundamental signature molecules of host defence explains why virtually all species of life produces host defence peptides in moderate to high concentrations. More than 1000 naturally occurring host defence peptides have been described to date, and the majority of these are covered in databases for eukaryotic host defence peptides, e.g., the site at the University of Trieste (http://www.bbcm.units.it/~tossi/pag1.htm) and the AMPer site (http://cnbi2.ca/cgi-bin/amp.pl) [33]. Despite their widespread distribution, these peptides are typically short (12 to 50 amino acids), carrying a net positive charge (+ 2 to + 9) due to excess basic arginine and/or lysine residues, and contain up to 50% hydrophobic amino acids. They are generally divided into four structural categories (*i.e.,* β-sheets, α-helices, loop- and extended-structures) (Figure 2), creating amphiphilic conformations upon interactions with lipid membranes, enabling membrane damage and/or penetration.

**Figure 3.**
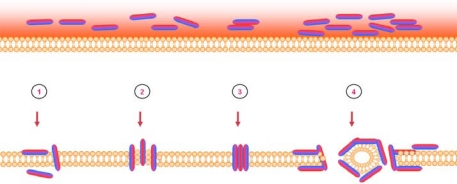
Mechanism of action of antimicrobial peptides targeting bacterial lipid membranes. The bacterial membrane is represented by the orange lipid bilayer with peptides illustrated as dual colored cylinders, with a hydrophilic region (red) and a hydrophobic region (blue). The arrangement of peptides on the surface of the lipid layer is hypothesized to drive four different membrane permeabilization mechanisms: the “aggregate” model **(1)**, the “torodal pore” model **(2)**, the “barrel-stave” model **(3)** and the “carpet” model **(4)**.

Initially cationic host defence peptides were investigated due to their direct ability to kill multidrug-resistant bacteria through extremely rapid mechanisms engaging and inhibiting multiple targets [34]. It was quickly realized that these peptides also carried a lytic ability, perforating bacterial membranes through “aggregate” [35], “toroidal pore” [36], “barrel-stave” [37], or “carpet” [38] mechanistic models (Figure 3). This naturally fuelled research investigating the peptides potential to lyse viral envelopes, as well. Later studies have also demonstrated the ability of many peptides (e.g., PR39, LL-37, LfcinB) to translocate across the cytoplasmic membrane of human cells while others are constitutively produced and stored as precursors inside host cell vacuoles [39–41]. Cellular uptake of these HDPs can result in gene/protein stimulation influencing host cell antiviral mechanisms [42], or might block viral gene/protein expression [43].

## 4. Antiviral activity of HDPs

Shortly after the discovery of the antibacterial potential of HDPs, it was confirmed that members from all four structural classes of HDPs also demonstrated the ability to inhibit viral infection, primarily affecting enveloped RNA and DNA viruses. Later it has been confirmed that viral infection with non-enveloped viruses, like adenovirus, feline calicivirus and echovirus 6 also can be inhibited [49–52]. Specifically for HSV, it was demonstrated that some α-helical peptides, *i.e.,* cecropins, clavanins, and the human cathelicidin LL-37, caused minimal or no viral inactivation [53–55], while other α-helical peptides like magainins, dermaseptin and melittin, demonstrated quite potent anti-HSV activity (Table 1) [55–58]. Conversely, β-sheet peptides like defensins, tachyplesin, protegrins and the β-turn peptide lactoferricin, have all shown high activity towards HSV (Table 1) [55,59–64]. Consequently, it appears impossible to predict peptide antiviral activity based on the primary or secondary structure of the peptides. However, the antiviral mechanism appears to be related to the viral adsorption and entry process [58], or to a direct effect on the viral envelope [56,65] indicating that the peptides ability to form stable amphiphilic conformation plays a crucial role for their antiviral activity.

## 5. Structural requirements for HDP antiviral activity

Several groups have made synthetic analogues of several naturally occurring HDPs to generate a firmer understanding of the structural requirements for peptide antiviral activity by changing chemical parameters such as charge and aromatic amino acids, as peptide antiviral activity often is correlated with both high cationic- and amphiphilic potential [55,60,66,67]. Other characteristics such as hydrophobicity, have been investigated in a hybrid peptide of cecropin A and magainin-2 [68], while D- or L-amino acids substitutions have been studied on a set of θ-defensins [64].

Substitution analysis studies have been reported for several different peptide classes, *i.e.,* the β-looped lactoferricin, an artificial α-helical peptide and the rigid cyclic θ-defensin. In the lactoferricin study, the relationship between HSV activity and peptide net charge and aromatic amino acids were investigated. The authors concluded that the spatial positioning of the charged residues were crucial for a potent peptide antiviral activity [61] while the aromatic residues were of less importance for anti-HSV activity [69]. The importance of a specific spatial presentation of the charged residues were reinforced by results from the substitution analysis study on the synthetic α-helical peptides [66,69]. These findings were also in accordance with results from Giansanti *et al.* [70] and Yasin *et al.* [64], which in separate studies on lactoferrin/transferrin derived peptides and θ-defensin analogues, respectively, concluded that the spatial conformation and presentation of positively charged residues were critical, but not sufficient for antiviral activity [64,70]. There are also results indicating that the rigidity of the peptides may contribute to the antiviral activity. Both lactoferricin and polyphemusin consist of β-structures stabilized by one and two internal disulfide bridges, respectively, and results have demonstrated that these stabilizing disulfide bridges are crucial for the peptides antiviral activity [61,67,82] (Figure 2, Table 1). However, a comparative study of peptides which clearly adopt different secondary structures have demonstrated that many of these peptides possess analogous antiviral modes of action, indicating that interaction with their target are independent of their structure [61,66]. The most likely explanation is that these HDPs are prone to adopt amphipathic conformations, which we have proposed are intrinsic to their antiviral activity.

## 6. Mode of action – blocking of viral entry by heparan sulfate interaction

Glycosaminoglycans are negatively charged molecules found in all tissue types: in intracellular granules, extracellular matrix, and on the cell surface [86–88]. As a result of their chemical composition, these molecules facilitate an interaction with almost anything carrying a slight cationic charge including small cations, proteins and peptides [89–95]. Heparan sulfate is the most important glycosaminoglycan and it has been demonstrated to serve as an important attachment receptor for several viruses including HSV [96,97]. Thus, it has been hypothesized that blocking of heparan sulfate can reduce viral attachment and consequently decrease the severity of the infection [10,11]. This has later been confirmed by using recombinant cells deficient in heparan sulfate expression, where it was demonstrated that the susceptibility to HSV infection was knocked down by 80% [12]. Similarly, by enzymatic removal of cellular glycosaminoglycans, it has also been demonstrated that HSV cell-to-cell spread, a viral mechanism used by HSV to evade the hosts’ immune regime, could be decreased [98], thus increasing the value of a potential drug targeting glycosaminoglycans. Inhibition of heparan sulfate biosynthesis (e.g., heparin, heparinase I and sodium chlorate) has also demonstrated the ability to inhibit human Cytomegalovirus infection in a dose-dependent manner [99]. Additionally, glycosaminoglycans have also been shown to be of major importance for HIV entry and replication [100], although they seem to be less important for HIV attachment to the host cell. Several HDPs, like human α-defensin, LL-37, magainin, bovine and human lactoferricin, have all been shown to interact with different glycosaminoglycans [101–105]. The sequence and structural diversity in these peptides suggest that the critical factor driving the interaction with glycosaminoglycan is the position of charged residues in the secondary structure. This is in accordance with the structural peptide studies correlating peptide affinity for heparan sulfate and anti-HSV activity, demonstrating that the affinity for heparan sulfate is only partly correlated with the peptides’ net charge [61,66]. In addition, it has been demonstrated that peptide analogues with arginine residues had a significantly higher affinity for glycosaminoglycan than comparable peptide analogues substituted with lysine [66,106–108].

HDP targeting of heparan sulfate is further supported by results from studies where peptide and virus were pre-incubated prior to infection. These studies demonstrate no increase in the peptide antiviral activity, confirming that cellular heparan sulfate was the primary target for these HDPs and not the viral interaction partner (*i.e.,* gC and/or gB) [39]. It should also be mentioned that HDPs exhibit different protective effects against HSV-1 and HSV-2, probably attributable to the combined effects of the peptides’ primary and secondary structures [59,61,66,109], something that has also been reported for other polycationic and even for polyanionic compounds [110,111]. Naturally, these differences may also reflect on the viral specificity of particular receptor molecules, and the differential ability of the peptides to interact with the different viral receptors.

## 7. Blocking of viral entry and cell-to-cell spread

Direct interaction between the HDPs and the glycoproteins in the viral envelope has been proposed to influence the viral attachment and entry. However, not many of the traditional peptides have been confirmed to actually possess this ability, though it has been proposed that dermaseptin can demonstrate anti-HSV activity through blocking of the attachment/adsorption/fusion phase of the HSV infection [58]. It has also been demonstrated that human neutrophil peptide (HNP)-1 (an α-defensin) neutralizes HSV-1 in a time-, temperature- and pH- dependent manner, in a process which is also antagonized by serum [60]. By pre-incubation of HNP-1 and HSV-2 prior to infection, it has been demonstrated that the infection can be reduced by >98%, while pre-treatment of the host cells with HNP-1 had no obvious effect on the anti-HSV activity. As the peptide still prevented viral entry, though not through competition for cellular heparan sulfate interaction, it is indicated that viral glycoproteins involved in membrane fusion may be the target [63]. Similarly, it has been demonstrated that θ-defensin (retrocyclin 2) directly binds HSV-2 glycoprotein B with high affinity, thus protecting the cells from HSV-2 infection [64]. Another possible interaction between the HDPs and the viral particle is through a peptide-lipid interaction, as HDPs are known for their ability to interact with lipid membranes, causing conformational changes leading to destabilization of the bacterial membrane, translocation of HDP, pore formation or lysis [112,113]. Indolicidin and dermaseptin is known to directly inactivate HIV-1 through membrane/envelope disruption [65,114]. Similar membrane disruption has been observed on vesicular stomatitis virus (VSV) after exposure to tachyplesin-1. This viral destruction is concentration-, time- and temperature- dependent, and electron micrographs of the tachyplesin-1 treated VSV particles showed naked and damaged virions, confirming the direct effect of the peptide on the viral envelope. Interestingly, treatment of HSV-1 and HSV-2 with tachyplesin-1 demonstrated no such effect on the viral envelope [115], indicating that the herpes envelope is relatively resistant to peptide damage. Experiments with cecropin, magainin, and lactoferricin are supporting this by demonstrating no direct peptide damage on the HSV envelope [57]. Electron micrographs of bovine lactoferricin exposed HSV also demonstrate a lack of direct interactions between the peptide and the viral particle (Jenssen, H; Unpublished results).

Cell-to-cell spread of HSV is one of the important mechanisms utilized by this virus to reduce cellular damage, consequently avoiding the initiation of a massive inflammatory response and silently escaping the immune system. It is well documented that several HDPs can reduce the cell-to-cell spread of HSV. For example, studies with rabbit α-defensin NP-1 and bovine lactoferricin, have demonstrated that these peptides inhibit both primary entry and cell-to-cell spread of HSV [39,63,66]. However, HDPs acting as inhibitors that only reduce part of the viral entry or spread will probably have limited success from a clinical point of view.

Continued discovery of new HSV entry receptors as reported by Perez *et al.* (*i.e.,* human B5 protein) will complicate the design of novel anti-HSV drugs [116]. Though this new receptor can be blocked by binding of an α-helical peptide in a coiled-coil formation [117] and probably also by other α-helical cationic peptides [66] effectively, there is hardly a valid alternative for completely blocking HSV entry unless a cocktail approach targeting all the attachment and entry receptors is adopted for drug design.

## 8. Intracellular targets and host cell stimulation

It is widely acknowledged that direct antimicrobial activities of HDPs are strongly antagonized by physiological salt concentrations, *i.e.,* 100mM monovalent- and 2mM divalent cations (as well as polyanions like glycosaminoglycans) [118]. Salts may also influence the peptide structure and consequently their association with anionic cell molecules, thus affecting their activity [119]. In addition to this, it has been demonstrated that the antiviral mode of action of α-defensin is serum dependent [120]. Together, this argues for the hypothesis that the systemic effects found with many HDPs are primarily a result of their immunomodulatory properties and to a lesser extent the peptides direct antimicrobial activity.

Rapid intracellular localization of the HDPs may support this hypothesis, and transmission electron microscopy studies have confirmed intracellular translocation of lactoferricin [39,98]. Bovine lactoferricin was also able to enter glycosaminoglycan-deficient cells in an energy-independent manner [39]. Similar experiments by Futaki *et al.* indicate that this mechanism is dependent on arginine content, a known feature of nuclear localization signals [121–123]. Human lactoferricin has multiple arginine residues [124] which probably contributes to the shuttling of this peptide into the nucleus where it can bind DNA. Due to many peptides’ abilities to interact with DNA, one might speculate that this directly influences viral nucleic acid synthesis, as has been shown for polyphemusin T22 and lactoferrin [125–128]. Consistent with this, the antiviral peptides LL-37 and indolicidin can both act as nuclear localization signals to translocate anti-sense nucleic acids [129].

Conversely, it is also known that HDPs have immunomodulatory activities which include the up-regulation of interferons and chemokines [118,120,130,131]. Thus many antiviral peptides might exert their activities in part by stimulating the antiviral immune system of the host cell. In addition to stimulating host cell responses, HDPs may also interfere with viral-triggered processes in the target cell. For example, it has been demonstrated that transport of HSV-2 tegument protein VP16 to the cell nucleus and consequent expression of ICP4 effectively are blocked in the presence of rabbit α-defensin (NP-1) [63]. Taken together, this prompted us to hypothesize that the most promising mechanism of HDPs in antiviral therapy is probably through stimulation of the host immune system as a mode of promoting pathogen clearance rather than direct blocking of viral attachment, entry and/or spread (Figure 1).

However, targeting the host immune system as a strategy for microbial intervention is not solely a strategically ingenious idea; it may also have a very devastating outcome, due to the extremely delicate balance between pro- and anti- inflammatory reactions. Thus, any type of immunotherapy should be initiated with extreme caution. It is also well documented that immune-suppressive chemicals and irradiation treatment will leave the patients vulnerable to new infections [132]. Additionally, there are also potential side effects with immune modulating drugs, like an inappropriate dysregulation of the immune system causing either sepsis or a cytokine storm [133]. In addition, HDPs like human β-defensin-2 and LL-37 can both induce histamine release [134] and many HDP derived peptides have demonstrated the ability to trigger apoptosis, or programmed cell death. It has also been suggested that over-expression of certain HDPs, like LL-37 in the skin will lead to an uncontrolled interferon response that will initiate the autoimmune skin inflammation in psoriasis patients [135]. While in patients suffering from a chronic inflammation of the intestine (Crohn’s disease) [136], it has been demonstrated that the epithelial cells lining the intestine are deficient in secreting β-defensins-2 and -3 [137,138] and LL-37 [139], resulting in an elevated level of luminal bacteria, thus allowing the natural microbial flora to trigger inflammation [140].

Despite these challenges, there are also several advantages in targeting the host innate immune system with HDP derivatives. The host immune mechanism is very general with broad spectrum activity. Thus, by optimizing a drug for immune modulation, it is likely that the drug will have a similar broad spectrum potential. As the pathogen itself is not targeted, there is very little chance of resistance development. HDP derivatives are generally well tolerated due to their chemical nature, though questions have been raised regarding the peptides’ poor pharmacokinetics profile. However strategies like N-methylation and pegylation have demonstrated the potential of significantly improving the oral bioavailability, metabolic stability and intestinal permeability of peptide drugs [141–143].

## 9. Adjuvant potential of HDP derived peptides

Vaccination dates back more than two hundred years to when Edward Jenner successfully vaccinated his patients against smallpox virus through exposure to cowpox virus, and the technique has since proven extremely effective as a public health initiative, reducing the devastating magnitude of several infectious diseases. However, there are only efficient vaccines against a handful of the human infectious diseases. In light of the immune stimulating role of natural HDPs and the ability of human cathelicidin (LL-37) to modulate dendritic cell differentiation and induced polarization, it has been proposed that HDPs may serve as novel adjuvants [144] in the production of more effective vaccines.

Traditional adjuvants have the tendency to cause undesirable injection site reactions as it is believed that it is necessary to retain the antigen at the site of injection as a depot to achieve an enhanced immune reaction [145]. As an example, vaccine development against respiratory syncytial virus (RSV) has been a struggle as the traditional adjuvants also have had a tendency to cause a Th2 biased immune response [146,147]. However, by reformulating the recombinant RSV fusion protein (DeltaF) with CpG oligodeozynucleotides, polyphosphazene and a HDP (indolicidin), it has been demonstrated that immunized mice developed a significantly higher level of RSV neutralizing antibodies compared to mice immunized with recombinant DeltaF alone [148]. Physical characterization of these vaccines has also indicated that the formulated vaccine demonstrated increased immunogenicity due to complex formation between the antigen and the adjuvant [149]. The adjuvant-antigen complex formation was rapid and appears to be less dependent on the peptide sequence, indicating that this process is relatively nonspecific. However, by testing several synthetic HDP derived peptides, it has been demonstrated that the increased immune response of these adjuvant complexes are peptide sequence specific [150,151]. Similar complex formation has also been alluded to in two separate vaccine formulation studies using artificial cationic peptides. In these studies, it was suggested that the peptides formed a transient depot at the site of injection, enhancing the association of antigen with antigen presenting cells [152,153]. However, it should be mentioned that one of these peptides, KLK-L_5_-KLK, also has been demonstrated to cause membrane rearrangement [154], facilitating uptake of oligonucleotides into the cellular compartments [155]. Thus, it is fair to conclude that HDPs as a vaccine adjuvant carries a very interesting potential, though the current understanding of the mechanism behind this adjuvant activity is somewhat limited.

## 10. Clinical potential of HDPs as antiviral therapeutics

Host defence peptides are an interesting class of agents with under-explored potential as antiviral therapeutics. Pioneer work exploring antiviral properties of naturally occurring HDPs reported interesting and promising results for several classes of peptides against numerous subfamilies of both naked and enveloped viruses. Despite the possibility of success from this work minor advancement has occurred beyond funneling these compounds into pre-clinical development. A handful of biotech companies, e.g., Magainin Pharmaceuticals (now Genaera; http://www.genaera.com/), Micrologix (now Migenix; http://www.migenix.com/) and IntraBiotics Pharmaceuticals (http://www.intrabiotics.com/) attempted to drive HDPs through clinical trial testing, but the majority of these pioneer projects were terminated after decades of struggle.

Peptides are traditionally viewed as very costly to manufacture, which has probably limited their potential for clinical exploration and use. However, a convergent strategy of synthesizing peptides through selection and assembly of peptide segments has proven to be useful in reducing cost and enabling large scale production for the commercial market. The best example of this is Roche (http://www.roche.com/index.htm) and Trimeris’ (http://www.trimeris.com/) joint success in large scale production of the HIV fusion inhibitor, T-20 (Efuvirtide, Fuzeon), by solid- and solution-phase hybrid synthesis [156,157]. T-20 is a 36 amino acid peptide that binds to a region of the HIV envelope glycoprotein gp41, thus, it is capable of blocking viral entry into CD4^+^ T-cells. The drug is currently only licensed for use in patients for whom other HIV medications are losing effectiveness, due in part to the limited annual production capacity (3.7 metric tons in 2005 providing treatment for ∼47,000 patients). Still, a more viable business strategy is probably the biotechnological approaches taken by Novozymes (Bagsvœrd, Denmark, http://www.novozymes.com/en), using a recombinant fungal expression platform for large scale production of plectasin with therapeutic purity in compliance with good manufacturing practices [44]. Plectasin is a 40 amino acid defensin-like peptide (Table 2) and a derivative of this (NZ2114) is currently being launched into pre-clinical development in collaboration between Novozymes and Sanofi-Aventis (Bridgewater, NJ, USA). Though NZ2114 is being investigated for its potential as a direct Gram-positive antimicrobial [158], this study may also encourage introduction of other defensin-like peptides into the clinic.

**Figure 4.**
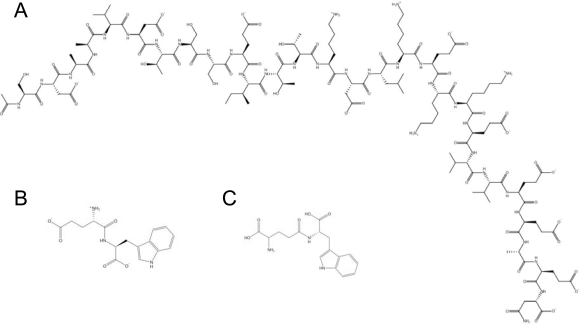
Antiviral peptides in clinical trials. **(A)** Zadaxin®, **(B)** IM862 and **(C)** SCV-07 are all compounds which have demonstrated promising results as immune modulators for treatment of patients with chronic hepatitis C virus infections.

Despite the limited clinical success of the cationic peptide drugs pioneering this field, several new ventures have subsequently been launched, with a strong involvement from academic labs worldwide. Consequently, though there are no current peptide drugs targeting herpes infections, there are several examples of viral diseases being treated with peptide drugs, illustrating the potential of driving these types of drugs into the clinic. For example, hepatitis B and C viruses are targeted with Thymosin α1 (ZADAXIN^®^, Thymalfasin), a 28 amino acid peptide originally isolated from calf thymus [159] (Figure 4, Table 2). It is currently being synthezised on solid phase by SciClone Pharmaceuticals International Ltd. (Causeway Bay, Hong Kong, China, http://www.scicloneinternational.com/) and approved for clinical use in more than 30 countries. Thymosin α1 enhances T_h_1 cytokine production along with T cell differentiation and maturation, augmenting the involvement of a specific T helper cell response in antiviral defence [160]. Another peptide being developed for hepatitis C virus treatment is the di-peptide (L-Glu-L-Trp, IM862) isolated from the calf thymic peptide complex Thymalin [161] (Figure 4, Table 2). Implicit Biosciences (Brisbane, QLD, Australia) is currently pursuing a sodium carboxylated salt of this peptide (Oglufanide disodium) in Phase IIa clinical trials. IM862 is known to have immunomodulatory properties [162] and it has also recently been demonstrated that lipidation of IM862 will increase the bioavailability of the drug [163]. Similarly, a synthetic analogue of IM862 named SCV-07 (gamma-D-glutamyl-L-tryptophan) (Figure 4, Table 2) has also been proven to have immune stimulating properties on T_h_1 cells, which are essential for clearance of viral infections. Recently, SciClone Pharmaceuticals International Ltd. reported positive results from Phase IIa clinical studies using SCV-07 as a monotherapy in patients with chronic hepatitis C infection. It has also been demonstrated recently that SCV-07 can significantly reduce recurrent herpetic lesions when administered orally but not subcutaneously in a guinea pig model of recurrent genital herpes simplex virus 2 [164].

This clearly indicates the potential for broad spectrum activity of such immune modulatory peptide drugs. Increasing knowledge and a broader understanding of the underlying mechanisms in the immune system together with technological advancements will probably advance the development of novel HDPs as alternative anti-infective drugs and one day also enable use of peptide-derived drugs in the treatment of HSV related infections.

## 11. Conclusion

We are rapidly approaching a crossroad where previously successful drug regimes are starting to fail and drug-resistant herpes strains are appearing. Thus, it is time to pursue alternative approaches and HDP-derived peptides may be a golden alternative. Many would argue that peptides acting as entry blockers of HSV for treatment are facing critical disadvantages as the virus easily adapts and is able to infect its host cells using several different receptors.

A more promising strategy would probably be to tailor peptide drugs promoting host cell immune responses. As HSV infections, in most cases, are self-resolving, innate defence regulators are prone to accelerate this process, reducing the infection period, thus having a tremendous positive impact on the patient for a reduced chance of resistance development. However, the concept of using HDP-derived drugs for antiviral treatment is still in its infancy and will require continued research and implementation of new technology platforms, *i.e.,* bioinformatics, to identify targets and generate a better understanding of the intrinsically complex immune response cascades.

## Figures and Tables

**Table 1.** Selected examples of antiviral peptides.

**Peptide**	**Structure**	**Source**	**Virus**	**Primary amino acid sequence**	**Proposed antiviral mechanism**	**References**
Magainin	α-helix	Frog	HSV	GIG KFLHSA KKFG KAFVG EIMNS	Cellular target	[56,57,71]
Cecropin A1	α-helix	Insect	HSV	GWL KKIG KKI ERVGQHT RDATIQGLGVAQ QAANVAATA R	Cellular target	[57,72]
Melittin	α-helix	Bee	HSV	GIGAVL KVLTTGLPALISWI KRKRQQ	Cellular target	[55,57,73]
LL-37	α-helix	Human	HSV	LLG DFF RKS KEKIG KEF KRIVQ RI KDFL RNL VP RT ES	Weak viral inactivation	[55,74]
Brevinin-1	α-helix	Frog	HSV	FLPVLAGIAA KVVPALFC_1_KIT KKC_1_	Viral inactivation	[55,75]
θ defensin	Cyclic β-sheet	Primate / human	HSV	G_1_FC_2_RC_3_LC_4_RRGVC_4_RC_3_IC_2_T R_1_	Binds gB and blocks viral attachment	[64,76]
Defensin	β-sheet	Human / rabbit	HSV HCMV	MPC_1_SC_2_KKYC_3_DPW EVI DGSC_2_GLFNS KYIC_3_ C_1_REK	Interacts with HSV membrane/ glycoprotein and cellular target but not heparan sulfate Inactivates viral particle	[60,63,64,77,78]
Dermaseptin	β-sheet	Frog	HSV	ALW KTML KKLGTMALHAG KAALGAAA DT ISQGTQ	Activity at virus-cell interface	[58,79]
Tachyplesin	β-sheet	Horse shoe crab	HSV	KWC_1_F RVC_2_Y RGIC_2_Y RRC_1_R	Viral inactivation	[55,80]
Protegrin	β-sheet	Human / porcine	HSV	RGG RLC_1_YC_2_RRRFC_2_VC_1_VG R	Viral inactivation	[55,81]
Lactoferricin	β-turn	Human / bovine	HSV HCMV	F KC_1_RRWQW RM KKLGAPSITC_1_V RRAFA	Blocks heparan sulfate, but a secondary effect has also been indicated. Activity at virus-cell interface	[59,61,82–85]
Indolicidin	Extended	Bovine	HSV	ILPW KWPWWPW RR	Targets viral membrane / glycoprotein	[47,57]

Note: The designation HSV indicates either type 1 or 2 (HSV-1 or HSV-2), or both types; human cytomegalovirus (HCMV) is another member of the human herpes virus family. The primary amino acid compositions of these peptides are given in one letter code. Cysteines forming disulfide bonds (or N-terminal to C-terminal linking) are numbered with subscript numbers to indicate their pairing. Boldface indicates cationic (blue) and anionic (red) amino acid residues.

**Table 2.** Selected examples of immunomodulatory and antimicrobial peptides in clinical trials or developmental stages.

***Drug***	***Sequence***	***Description/Status/Results***	***Company & reference***
Plectasin NZ2114	GFGC_1_NGPWDEDDMQC_2_HN HC_3_KSIKGYKGGYC_1_AKGGF VC_2_KC_3_Y-COOH (Plectasin)	**Preclinical:** A variant of plectasin which has demonstrated potent Gram-positive effect in systemic pneumococcal and streptococcal infections.	Novozymes AS / Sanofi-Aventis (Bagsvœrd, Denmark), www.novozymes.com

Zadaxin® / Thymosin α1 / thymalfasin	AC-SDAAVDTSSEITTKDLK EKKEVVEEAEN-COOH	**Phase III / approved:** A 28-mer bovine HDP intended for directed therapy targeting chronic hepatitis B and C viruses. It promotes MHC class I expression, interleukin-2 and interferon-γ secretion, proliferation and activation of CD4 T_h_1, CD8, and NK cells. It also decreases T_h_2 cytokines (e.g., interleukin-4 and interleukin-10) that are counter productive to viral infections.	SciClone / Sigma-Tau (Foster City, CA, USA), www.hcvadvocate.org, www.scicloneinternational.com

Oglufanide disodium / IM862	EW-COOH	**Phase II:** A di-peptide for direct targeting chronic hepatitis C virus. Administration of an intranasal synthetic formulation of IM862 has demonstrated to reverses the suppression of the immune system.	Implicit Bioscience (Toowong, QLD, Australia), www.hcvadvocate.org

SCV-07	γ-D-glutamyl-L-tryptophan	**Phase IIa:** SCV-07 has demonstrated promising results as a mono-therapy in patients with chronic hepatitis C virus infection.	SciClone www.scicloneinternational.com

*Note:* Amino acid sequences are given in one-letter code. Cysteines forming disulfide bonds are numbered with subscripts to indicate their pairings. N- and C-terminal modifications are indicated before the hyphen in the front or after the hyphen at the end of the sequence.
